# The effect of measurement error of phenotypes on genome wide association studies

**DOI:** 10.1186/1471-2164-12-232

**Published:** 2011-05-12

**Authors:** William Barendse

**Affiliations:** 1 Cooperative Research Centre for Beef Genetic Technologies, CSIRO Livestock Industries, Queensland Bioscience Precinct, 306 Carmody Road, St. Lucia, Queensland, 4067, Australia

## Abstract

**Background:**

There is an unspoken assumption that imprecision of measurement of phenotypes will not have large systematic effects on the location of significant associations in a genome wide association study (GWAS). In this report, the effects of two independent measurements of the same trait, subcutaneous fat thickness, were examined in GWAS of 940 individuals.

**Results:**

The trait values obtained by two independent groups working to the same trait definition were correlated with r = 0.72. The allele effects obtained from the two analyses were only moderately correlated, with r = 0.53, and there was one significant (*P *< 0.0001) association in common to the two measurements. The correlation between allele effects was approximately equal to the square of the correlation between the trait measurements. An important quantitative trait locus (QTL) on BTA14 appeared to be shifted distally by 1 Mb along the chromosome. The divergence in GWAS was stronger with data coded into two discrete classes. Univariate trimming of the top and bottom 5% of data, a method used to control for erroneous trait values, decreased the similarity between the GWAS and increased the apparent shift of the QTL on BTA14. Stringent bivariate trimming of data, using only trait values that were similar to each other in the two data sets, substantially improved the correlation of trait values and allele effects in the GWAS, and showed evidence for two QTL on BTA14 separated by 1 Mb. Despite the reduction in sample size due to trimming, more SNP were significant. Using the mean of the two measurements of the trait was not as efficient as bivariate trimming.

**Conclusions:**

It is recommended that trait values in GWAS experiments be examined for repeatability before the experiment is performed. For traits that do not have high repeatability (r < 0.95), two or more independent measurements of the same trait should be obtained for all samples, and individuals genotyped that have highly correlated trait measurements.

## Background

It is usually assumed that a trait is measured with a degree of imprecision or error, but that the details of the measurement of a trait will not affect the results of a genome wide association study. Specifically, that 1) the particular details of how the trait is measured, 2) who measured it, and 3) whether the trait is subtly differently measured in a second sample will not i) materially affect the location of the most significant associations between a DNA marker and the trait of interest or ii) affect the confirmation of the results in a second sample, because it is assumed that the errors in measurement are not systematic. Genome wide association studies (GWAS) compare a large number of single nucleotide polymorphisms (SNPs) to a trait measurement and SNPs with strong associations are usually reported [1,2]. The effect of genotypic error is well known and there is a substantial amount of error checking of genotypes that usually occurs before the GWAS is performed. Thereafter, a second sample is used for confirmation usually measured in the same way but often by a different set of phenotypers, and there may be subtle differences in the way the phenotypes were measured. The lack of confirmation of some of the associations is usually ascribed to differences in some aspect of the genetics of the trait or aspects of the sample, and lack of confirmation is most generally ascribed to false discovery due to the large number of tests performed or insufficient power in the original GWAS [3,4]. For some traits, particularly ones that rely upon qualitative diagnosis, care is taken to ensure that the same diagnostic criteria are used [5], but there is always the possibility that different individuals will interpret the criteria in their own way, leading to heterogeneity in classifying individuals from one sample to the next. With quantitative traits, such as a height or weight measurement, this is usually not thought to be an important source of variability, but many quantitative traits are not automatically sampled by machine nor have precise and unambiguous guidelines for measurement, and alternative measurements of a trait may have correlations r < 0.5 [6].

Two measurements of subcutaneous fat thickness were collected on the same animals by two groups working independently [7] and this provided the opportunity to examine the effects of measurement error of phenotypes on the results of a GWAS. Of course, subcutaneous fat thickness is also of intrinsic interest in biological studies in many mammalian species because it is linked to the overall fatness of an animal or how well it is doing in a particular environment, to onset of puberty, and is easily measured [8,9]. The thickness of the fat layer in these studies was measured manually using a ruler with 1 mm gradations adjacent to the crest of the 3^rd ^sacral vertebra, but as fat layers are not uniformly thick there is clearly scope for variation in measurements performed by different individuals. These animals had been genotyped for a GWAS of body composition [10] and one of the fat thickness measurements had been analysed along with estimates of growth and food efficiency. To determine how successful confirmation would be in future studies of the same trait, and to evaluate how successful others would be in confirming our findings, the likely importance of such imprecision was investigated by comparing the results of the GWAS for this trait to a GWAS performed using the unpublished second measurement of subcutaneous fat thickness.

This analysis took previously collected genotypic data, additional unpublished phenotypes and then reanalysed the entire data set. The expectation was that there would not be systematic differences in the two measurements, that the underlying genetic basis for fat thickness would be evident in both GWAS, and that the differences in trait values might merely affect the degree of significance of the most significant SNP associations, which would largely be in common. The data were trimmed in two ways to remove outliers to determine whether this would be successful in controlling the differences between the GWAS. The fat thickness data were also coded as a threshold trait to determine how important such measurement error might be for discrete traits, where individuals may be coded as affected or unaffected.

## Results

**Figure 1:**
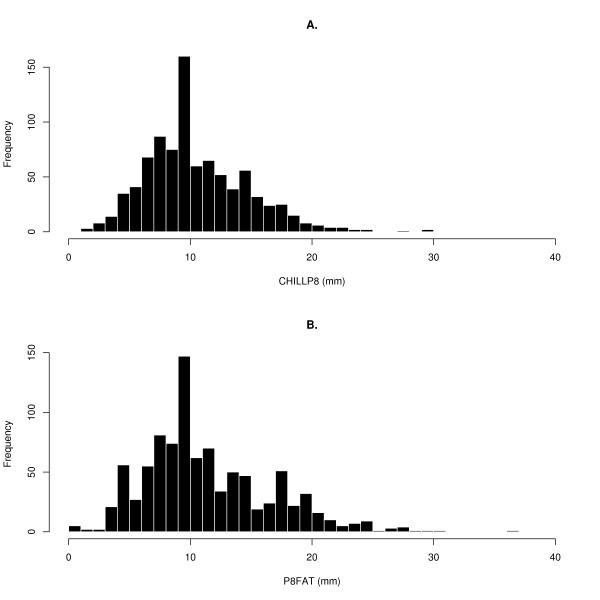
**Distribution of trait measurements in the sample**. A. Histogram of CHILLP8 fat thickness for animals in the GWAS sample. Note the excess frequency at 10 mm, B. Histogram of P8FAT thickness for animals in the GWAS sample, note the excess frequency at 10 mm and that the distribution appears less smooth than the CHILLP8 distribution.

**Figure 2:**
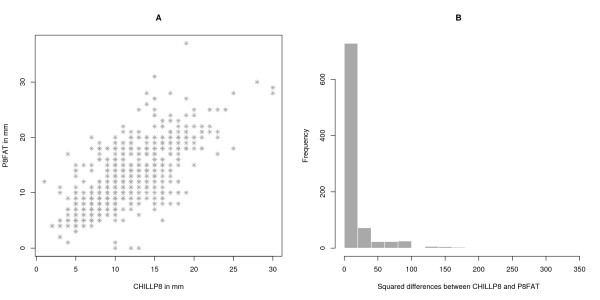
**Deviation between the two measurements of the trait**. A. Bivariate plot of CHILLP8 and P8FAT for animals in this study, this plot does not show the bivariate density of the two measurements because all the measurements are integers so they stack one on top of the other. B. Histogram of squared differences between CHILLP8 and P8FAT measurements for the GWAS sample, note that the difference between the vast majority of measurements is < 5 mm.

The two subcutaneous fat thickness measurements for the animals that were genotyped differed by an average of 1.10 mm (s.e.m. = 0.12), the correlation was r = 0.72 between them, and the regression coefficient of CHILLP8 on P8FAT was *b *= 0.89, s.e. = 0.03 (Table 1). In all untrimmed data sets the P8FAT measurement had a larger range and greater variance than the CHILLP8 measurement. The histograms for the CHILLP8 and P8FAT distribution (Figures 1A &1B) both have a longer right tail than left tail and are similar in shape but the CHILLP8 histogram appears smoother than the P8FAT histogram. Most measurements were similar to each other but a substantial number of individuals showed highly divergent results (Figures 2A &2B). Univariate trimming, by removing the top and bottom 5% of values, reduced the range substantially for CHILLP8 and P8FAT, but did not improve the correlation between traits, which reduced to r = 0.61. Bivariate trimming substantially improved the correlation between measurements but left the range and variance relatively intact. Using the mean of the two P8 measurements resulted in correlations that were similar to that obtained with bivariate trimming at diff1 < 4 between the two measurements, but resulted in a larger sample for analysis.

**Table 1:** Characteristics of the subcutaneous fat thickness data with different types of trimming

Trait	Sample	n	mean (mm)	**s.d**.	**s.e.m**.	**C.V**.	range (mm)	***r***^**B**^
CHILLP8^A^	GWAS	888	10.99	4.18	0.14	0.38	1-30	-
P8FAT ^A^	GWAS	940	11.89	5.21	0.17	0.44	0-37	0.72
P8MEAN ^A^	GWAS	888	11.55	4.34	0.15	0.38	2.5-29.5	0.91/0.94 ^C^
CHILLP8	trim10%	799	10.80	3.22	0.11	0.30	5-18	-
P8FAT	trim10%	846	11.63	4.10	0.14	0.35	5-21	0.61 ^D^
CHILLP8	diff1 < 36	800	10.92	4.16	0.15	0.38	2-30	-
P8FAT	diff1 < 36	800	11.56	4.62	0.16	0.40	1-30	0.85
CHILLP8	diff1 < 4	564	10.46	4.09	0.17	0.39	2-30	-
P8FAT	diff1 < 4	564	10.68	4.23	0.18	0.40	2-30	0.95
CHILLP8	all	8,139	10.75	5.05	0.06	0.47	0-38	-
P8FAT	all	8,653	11.63	5.65	0.06	0.49	0-42	0.81

^A ^Subcutaneous fat thickness measured in millimetres aligned with the crest of the 3^rd ^sacral vertebra by two independent groups, with P8MEAN the average between the two values

^B ^Correlation between CHILLP8 and P8FAT for the same method of data trimming

^C ^Correlation between P8MEAN to CHILLP8 and P8FAT respectively

^D ^For samples in common for the two univariate trimmed data sets

### P8FAT GWAS and difference to the CHILLP8 GWAS

**Figure 3:**
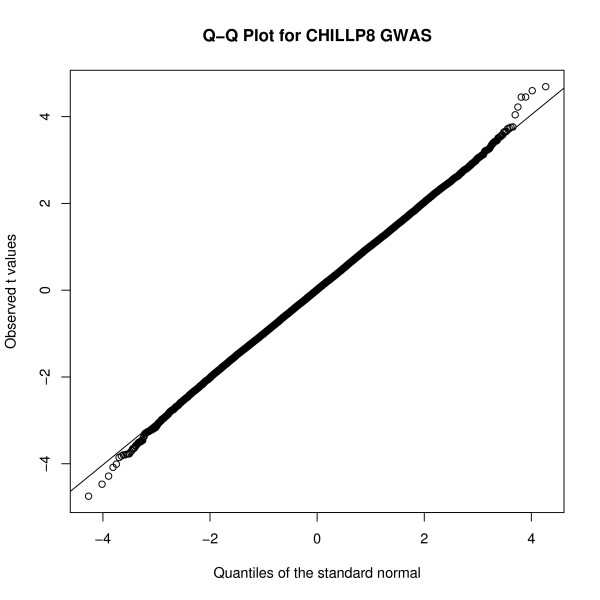
**The Q-Q plot of t-values in the CHILLP8 GWAS**. The quantile-quantile plot of the observed distribution of the t-values for the GWAS of CHILLP8 compared to the theoretical distribution. The plot represents at least 50 thousand data points. Points at the extreme of the observed distribution show values that were larger than expected.

**Figure 4:**
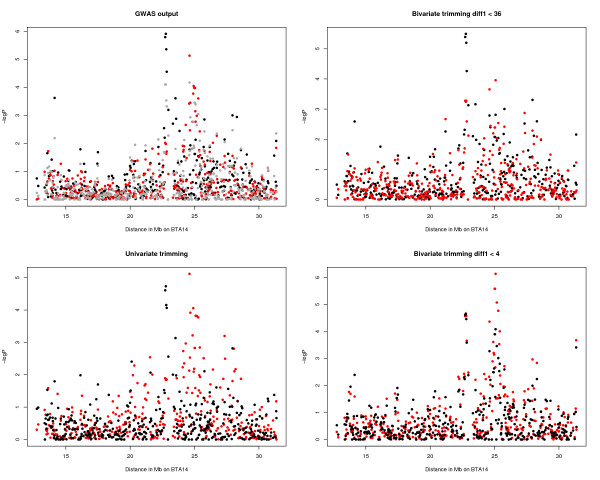
**Evidence for QTLs on BTA14 under different kinds and amounts of data trimming**. Manhattan plots of SNP associations to CHILLP8 (black) and P8FAT (red) plotted against distance along part of chromosome BTA14 in Mb. The four panels show the original GWAS data, which also includes P8MEAN (dark grey), the univariate trimmed data excluding the top and bottom 5% of values, the bivariate trimmed data with the threshold set at diff1 < 36, and the bivariate trimmed data with the threshold set at diff1 < 4.

**Figure 5:**
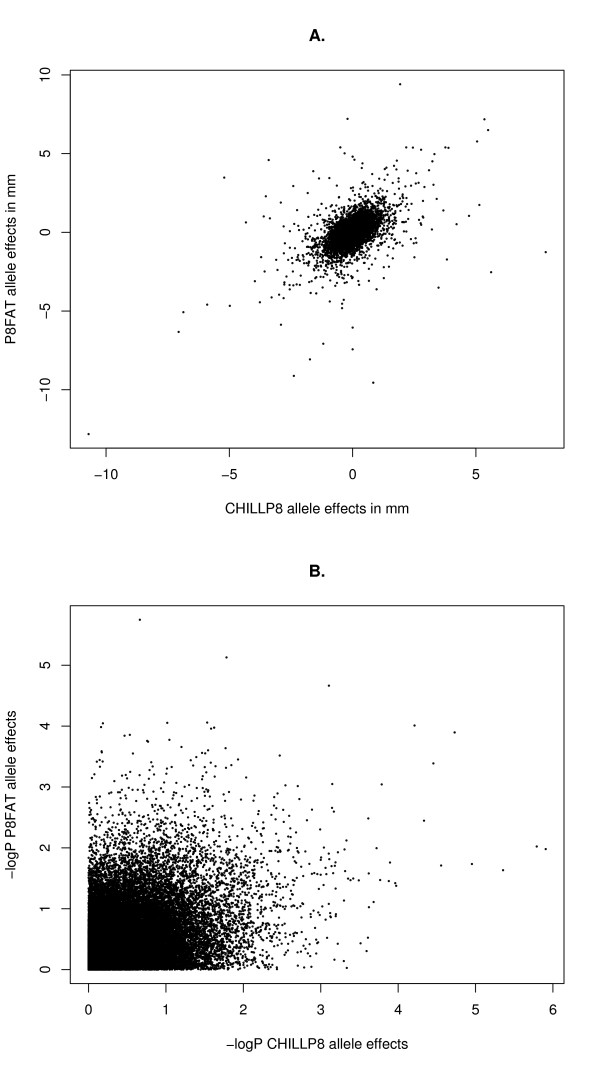
**The divergence between allele effects and their significance in the GWAS of the two trait measurements**. A. Bivariate plot of allele effects of CHILLP8 (x-axis) and P8FAT (y-axis), the relationship has a correlation of r = 0.53, B. Bivariate plot of -log*P *values of CHILLP8 (x-axis) and P8FAT (y-axis), the relationship has a correlation of r = 0.36.

The GWAS of the P8FAT measurement found 71 SNPs significantly (*P *< 0.001) associated to the trait, which represents a false positive rate (FPR) = 76% (Table 2). This is a slightly lower FPR than that found for the GWAS of the CHILLP8 measurement, which reported 63 SNPs significantly (*P *< 0.001) associated to that trait measurement, and FPR = 86%. These associations showed a distinct excess of large t-test values compared to the theoretical expectation, consistent with the discovery of real associations (Figure 3). There were 7 significant (*P *< 0.001) SNP in common (Table 2). The largest aggregation of significant SNP associations in the P8FAT GWAS was 10 SNPs out of 20 with *P *< 0.001 at BTA14:25 Mb, in a 705.9 kb region that includes the genes *NSMAF *and *TOX*. The largest aggregation of significant SNP association in the CHILLP8 GWAS was 6 SNPs out of 11 with *P *< 0.001 on BTA14:23 Mb, in a 799.1 kb region that included the genes *XKR4, TMEM68, TGS1, LYN, RPS20, LOC787404, MOS, PLAG1, CHCHD7, SDR16C5*, and *PENK*. Six of these 31 SNP on BTA14 associated to either measurement were significant (*P *< 0.0001) including BTB-01530836, which had *P *= 1.2 × 10^-6 ^(Table 3). These two aggregations of SNPs are adjacent to each other but do not overlap and are separated by 1.06 Mb (Figure 4). This QTL region on BTA14 appears to be substantially stronger than all others for this trait although its exact location appears to shift depending on the trait measurement used. To summarise the differences in the GWAS, the correlation of allele effects was r = 0.53 and of -log*P *values was r = 0.36 (Figure 5). These correlations are substantially less than the correlation between measurements, and the correlation between the allele effects was approximately equal to the square of the correlation between the trait measurements (i.e., r = 0.72 gives r^2 ^= 0.52).

**Table 2:** Summary of genome wide associations for CHILLP8 and P8FAT under different kinds of trimming.

Trait	Type	N ** SNP**^**A**^	**FPR**^**B**^	Common Sig ** SNPs**^**C**^	***r***^**D**^	Top 5 **Chr**^**E**^	Sig ** %**^**F**^	Best ** region**^**G**^	**Gene(s) **^**H**^
CHILLP8	GWAS	63	84%	-	-	14, 7, 8, 1, 6	42.9%	BTA14:23	*XKR4 *to *PENK * including *PLAG1*
P8FAT	GWAS	71	76%	7	0.53	14, 9, 6, 8, 7	45.1%	BTA14:25	*NSMAF * *& TOX*
P8MEAN	GWAS	59	92%	22/23^I^	0.84/0.88^I^	14, 8, 6, 3, 13	55.9%	BTA14:23 & 25	*XKR4 *to *TOX*
CHILLP8	trim10%	57	95%	-	-	14, 1, 11, 3, 22	38.6%	BTA14:23	*XKR4 * to *PENK*
P8FAT	trim10%	66	82%	0	0.38	14, 9, 29, 11, 10	47.0%	BTA14:25	*NSMAF* * & TOX*
CHILLP8	diff < 36	53	100%	-	-	14, 8, 3, 18, 13	45.2%	BTA14:23 & 25	*XKR4 *to *TOX*
P8FAT	diff < 36	52	100%	10	0.75	14, 2, 3, 1, 8	42.3%	BTA14:23 & 25	*XKR4 *to *TOX*
CHILLP8	diff < 4	81	67%	-	-	14, 11, 1, 2, 7	48.1%	BTA14:23 & 25	*XKR4 *to *TOX*
P8FAT	diff < 4	98	55%	49	0.92	14, 11, 15, 2, 6	45.0%	BTA14:23 & 25	*XKR4 *to *TOX*

^A ^Number SNP significant at *P *< 0.001

^B ^False positive rate

^C ^Number of significant (*P *< 0.001) SNP in common between a CHILLP8 and P8FAT GWAS of the same type

^D ^Correlation between allele effects of GWAS of CHILLP8 and P8FAT for the same method of data trimming

^E ^Top 5 chromosomes with largest number of SNP significant at *P *< 0.001 in descending order of number of significant SNP

^F ^Percent of SNP with *P *< 0.001 on the top 5 chromosomes compared to all chromosomes

^G ^Genomic region with the largest number of SNP significant at *P *< 0.001

^H ^Gene(s) located to this genomic region

^I ^P8MEAN and CHILLP8 or P8FAT respectively

**Table 3:** Significant (*P *< 0.0001) SNP in the GWAS analyses of CHILLP8 and P8FAT

SNP		gene symbol		Chr		position (bp)		Allele		**f**^**A**^		*b * (mm)		**s.e**.		*P*
CHILLP8																
ARS-BFGL- NGS-97163		proximal SOX14		1		133446995		G		0.01		2.85		0.64		1.1 × 10^-5^
BTB- 00174922		proximal SEMA3D		4		36143304		C		0.98		-2.77		0.64		1.9 × 10^-5^
BTB- 00174955		proximal SEMA3D		4		36253391		G		0.98		-2.77		0.64		1.9 × 10^-5^
Hapmap55575- rs29016266		UNC5C		6		31006416		C		0.31		0.77		0.19		6.1 × 10^-5^
ARS-BFGL- NGS-100395		TNC		8		109649680		C		0.23		-0.81		0.20		4.6 × 10^-5^
BTB- 01530788		XKR4		14		22720373		C		0.21		1.06		0.22		1.6 × 10^-6^
BTB- 01530836		XKR4		14		22768980		G		0.79		-1.08		0.22		1.2 × 10^-6^
BTB- 00557585		XKR4		14		22803366		C		0.21		1.02		0.22		4.4 × 10^-5^
BTB- 00557532		distal XKR4		14		22838801		G		0.21		0.93		0.22		2.8 × 10^-5^
Hapmap53460- rs29027620		SUCLG2		22		34764990		G		0.41		-0.69		0.17		3.5 × 10^-5^
P8FAT																
Hapmap42233- BTA-49670		proximal LOC615631		1		83842132		C		0.83		-1.08		0.27		8.7 × 10-5
Hapmap50089- BTA-75090		DMC1		5		117300831		G		0.06		-2.12		0.44		1.8 × 10^-6^
ιHapmap55575- rs29016266		UNC5C		6		31006416		C		0.30		0.94		0.24		9.8 × 10^-5^
ARS-BFGL- NGS-26768		proximal ZNF474		7		30315940		G		0.56		-0.95		0.22		2.2 × 10^-5^
BTB- 00529060		TLE1		8		60156808		G		0.18		-1.21		0.31		8.8 × 10^-5^
Hapmap32434- BTC-011497		NSMAF		14		24607054		G		0.65		0.94		0.21		7.4 × 10^-6^
UA-IFASA- 7902		TOX		14		24933303		C		0.46		0.85		0.22		9.0 × 10^-5^

^A ^Allele frequency across the entire sample

### The effect of data trimming

Univariate trimming of the top and bottom 5% of data for CHILLP8 or P8FAT decreased the similarity between the two GWAS. The correlation of allele effects decreased to r = 0.38, a value similar to the square of the correlation between the CHILLP8 and P8FAT measurements after trimming the top and bottom 5% of measurements (r = 0.61 gives r^2 ^= 0.37). None of the significant (*P *< 0.001) SNPs were significant for both CHILLP8 and P8FAT (Table 2). The clusters of SNP on BTA14 were in different locations in the two GWAS, similar to that found in the full data set (Figure 4), with a slightly greater distance between the clusters. The most significant SNP in the CHILLP8 GWAS was now on BTA8:82 Mb (BTB-01733915) and the most significant SNP in the P8FAT GWAS was on BTA14:25 Mb (Hapmap32434-BTC-011497).

Bivariate trimming improved the correlation between allele effects for a GWAS of P8FAT and CHILLP8. Trimming at diff1 < 36 decreased the sample size by 140 and 88 respectively for the P8FAT and CHILLP8 datasets but increased the correlation of allele effects to r = 0.75, a value similar to the square of the correlation between the CHILLP8 and P8FAT measurements after trimming at diff1 < 36 (r = 0.85 gives r^2 ^= 0.72). The number of significant (*P *< 0.001) associations decreased for CHILLP8 and P8FAT, but there was an increase in the number of SNPs that were significant in common. The CHILLP8 and P8FAT GWAS showed two clusters of SNP, one at BTA14:23 and one at BTA14:25 Mb, although there were more SNP in the BTA14:23 Mb cluster than in the BTA14:25 Mb cluster for both GWAS (Figure 4).

Bivariate trimming at diff1 < 4 decreased the sample by 376 for P8FAT and 324 for CHILLP8 and not only increased the correlation between allele effects it reduced the FPR for the experiment and generated smaller p-values for the SNP. The correlation between allele effects between the GWAS increased to r = 0.92 which is approximately equal to the square of the correlation of the trait values trimmed at diff1 < 4 (r = 0.95 gives r^2 ^= 0.90). The number of significant SNP nearly doubled (Table 2), the number of significant SNP in common increased to 49 and there were 14 SNP in common at *P *< 0.0001. On BTA 14, each of the GWAS showed two clusters of SNP separated by approximately 1 Mb, with both GWAS showing SNP significant (*P *< 0.0001) in both clusters (Figure 4), and the SNP Hapmap25761-BTC-065280 (BTA14: 25081788) was associated with P8FAT with *P *= 7.4 × 10^-7^.

An alternative to trimming is to average the two trait measurements (P8MEAN), which may reduce the number of highly divergent measurements and preserve as high a sample size as possible. Overall, the correlation between CHILLP8 and the P8MEAN allele effects was r = 0.84 and between P8FAT and P8MEAN allele effects was r = 0.88. These are approximately equal to the square of the correlations between the trait measurements of CHILLP8 and P8MEAN and P8FAT and P8MEAN (r = 0.91 gives r^2 ^= 0.83 and r = 0.94 give r^2 ^= 0.88, respectively). The number of significant SNPs were relatively low (Table 2) but a large proportion were shared and 7 significant SNPs were shared between all three measurements. Importantly, there was evidence for both clusters of significant SNPs associated to P8MEAN on BTA14, however, there were fewer significant SNP in each cluster, and the *P*-values were not as small (Figure 4). Averaging the trait measurements was not as efficient as stringent bivariate trimming.

### The effect on discrete phenotypes

When the phenotypes were coded as affected or unaffected, depending upon whether they exceeded the +1 s.d. threshold above the mean, the effect of misclassification was greater on these results, but bivariate trimming was able to mitigate these effects substantially (Table 4). The correlation between allele effects for the full data set was r = 0.45 and increased to r = 0.68 for bivariate trimming at diff1 < 4. The percentage discordantly scored individuals declined from 14.5 to 5.3%. With discrete phenotypes the associations were more significant, and at a stringency of diff1 < 4 there were 236 significant (*P *< 0.001) SNPs in the CHILLP8 GWAS, 128 significant (*P *< 0.001) SNPs in the P8FAT GWAS. There were 22 significant (*P *< 0.001) SNPs common to both of the GWAS, four of these common SNPs were significant at the 1 × 10^-5 ^threshold, and one of these SNPs was significant at the 1 × 10^-7 ^threshold. Obviously, with discrete phenotypes it is possible to exclude all discrepantly coded individuals, and all +A and A+ individuals (Table 4) could be removed to give exactly the same output from a GWAS. After bivariate trimming at diff1 < 4, and then removal of the 20 discordant phenotypes, there were 4 SNPs that were significant at *P *< 1 × 10^-8 ^and 129 SNPs that were significant at *P *< 0.001, obtained with a sample of n = 359 animals.

**Table 4:** Effects on coding of affected and unaffected individuals at a threshold

	**Phenotype similarity**^**A**^					
Data set	++	+A	A+	AA	%^B^	r^C^
Full data	704	64	64	56	14.4	0.45
diff1 < 36	652	19	48	51	8.7	0.55
diff1 < 4	342	2	18	17	5.3	0.68

^A ^Numbers of individuals coded as unaffected (+) or affected (A) using a threshold of > +1 s.d. in the CHILLP8 and P8FAT data, respectively

^B ^Percentage of discrepant codes for both traits

^C ^Correlation of allele effects for the discretised CHILLP8 and P8FAT data

## Discussion

The error in measuring phenotypes had a strong effect on the profiles of significant SNP identified in the GWAS. There was a small overlap in the significant SNPs, the chromosomes with the largest number of significant SNPs were similar but not the same, and the region with the largest number of significant SNPs, occurring on BTA14 in both of the trait measurements, was shifted by at least 1 Mb towards the telomere. Follow up studies of this important QTL on BTA14 could have ended up wasting time studying a non-overlapping set of genes. Overall, the correlation in allele effects was moderate and was approximately equal to the square of the correlation between the trait measurements for all data sets. As the correlation between trait measurements declines below approximately r = 0.95 between duplicate trait measurements, the similarity between GWAS will decline rapidly. For qualitative traits using a threshold to determine whether an individual is affected, measurement error appeared to generate greater incompatibility between the GWAS, possibly because it is a more powerful statistical design. These results suggest that one would expect to find minimal overlap in the significant SNP from one study to the next if a different but correlated trait was used in a confirmation study, such as rib fat thickness or intramuscular fat percentage.

The attempts to control measurement error were not all equally successful, and even small amounts of measurement error still caused differences in the GWAS. Univariate trimming, which is generally used to remove obviously erroneous outliers, such as a 1,000 kg human, or a 90 kg peregrine falcon, made the comparison between GWAS worse. Nevertheless, despite the increased divergence between measurements, the QTL on BTA14 was still found in both GWAS, except that the disparity in the location of the QTL in each GWAS increased. Bivariate trimming, even at an equivalent of the removal of around 10% of the most divergent measurements, showed an improvement in similarity between the GWAS. At the most stringent level, the overall experimental statistics such as the FPR and the size of the p-values improved, so that smaller p-values were generated for the significant SNP despite the decrease in sample size. More importantly, for the QTL on BTA14, its location was clarified into two adjacent QTL, separated by 1 Mb. Using the mean of the two measurements helped to reduce phenotypic error, also showed two QTL separated by 1 Mb on BTA14, but the FPR was close to 100% and the p-values were substantially larger. This suggests that taking the average does not remove error, and it does not reduce error sufficiently that the errors will not affect a GWAS study. Nevertheless, even with the most stringent amounts of bivariate trimming, so that the trait measurements were very highly correlated, the list of significant SNP of the two GWAS did not coincide. This suggests that the deterministic methods used to analyse GWAS data are sensitive to small differences when mapping quantitative traits and every effort needs to be made to improve the precision of trait measurement, or to develop methods of analysis that are not so sensitive to error. It is therefore recommended that trait values in GWAS experiments be examined for repeatability before the experiment is performed. At present, duplicate, independent measures of the phenotype are not made for GWA studies, so routine bivariate trimming would not be possible for most data sets. For traits that do not have high repeatability (r < 0.95), two or more independent measurements of the same trait should be obtained for all samples, and individuals genotyped that have highly correlated trait measurements.

The major differences in the trait measurements in this study were that 1) a different group of individuals performed the measurement (accredited AUSMEAT inspectors versus trained meat scientists), and 2) the P8FAT measurement was performed about an hour after slaughter on a warm carcass whereas the CHILLP8 measurement was performed after the carcass had been refrigerated, and would have been measured within 24 hours of the P8FAT measurement. Although one cannot explicitly disentangle the effects of warm vs chiller and inspectors vs meat scientists, because the comparison was warm+inspectors vs cold+scientists, the differences between the mean CHILLP8 and P8FAT were ~ 1 mm and the regression coefficient of one measure on the other was nearly 1 in the entire sample (*b *= 0.90) with a small standard error of estimation (s.e. = 0.01), which suggests that overall, there was little shrinkage in the fat thickness. Furthermore, the plot of divergence suggests that most of the difference is due to strongly divergent measurements for a minority of samples, implicating operator error rather than systematic differences due to the chiller. Nevertheless, given these factors of when, where and by whom the trait was measured, these measurement differences may be at the extreme end of divergence of measurement of the same trait, and studies of other traits may not be as strongly affected. However, until other analyses of independently performed repeated measurements are obtained one cannot be sure that the results obtained here are atypical or that they will represent a general phenomenon.

More importantly, these results suggest that a wide variety of phenotypes may be subject to unreproducible results due to technical issues associated with phenotype measurement. In the measurement of fat thickness, fat layers are not uniformly thick and so slight differences in where an individual or group of scorers placed the ruler could potentially affect the measure obtained. Some phenotypes are more likely to be affected by such measurement problems. For example, metabolite or hormone concentrations may show diurnal or weekly cycles or may show different values in different assays [11]. A bone thickness or length will depend upon a landmark being identified and used consistently [12]. A waist measurement, defined as the region of smallest circumference, might be made at a different location in each individual, or if measured at a particular part of the abdomen might not be the smallest circumference [13]. These results also show that the effects of measurement accuracy are stronger on the coding of affected or unaffected status, such as when an individual passes a threshold. Traits that may be affected by such thresholds are schizophrenia or obesity.

These effects on GWAS studies yield sobering implications for genomic selection or phenotype prediction. Genomic selection or phenotype prediction uses LD between markers and traits in one study to predict the performance of a separate set of animals using their genotypes for the same panel of markers, usually a set of tens to hundreds of thousands of SNPs in a SNP array [14,15]. There is the implicit assumption in those studies that if large enough numbers of SNPs and large enough sample sizes of animals are used then high predictive accuracy will be obtained. Genomic selection depends for its success not only on the significance of the most significant SNPs but includes loci with minimal evidence for significance and even SNPs with non-significant effects into these models [16-19]. The results in this study show that evidence for larger QTL can appear to shift by 1 Mb along the chromosome, and the evidence for smaller QTL can disappear altogether, which will affect the list of SNP used in these predictions. In addition, genomic selection analyses may be affected by trait measurement error because even after the majority of discordant data have been removed the allele effects in the two GWAS still showed clear differences. These results suggest that the prediction accuracy of such genomic selection or prediction studies will only improve once better phenotypic measures are collected, either by double scoring the phenotypes or by more accurate machine based collection of precisely defined phenotypes.

## Conclusions

It is recommended that trait values in GWAS experiments be examined for repeatability before the experiment is performed. Wherever possible, independent scorers should collect the repeated data. For traits that do not have high repeatability (r < 0.95), two or more independent measurements of the same trait should be obtained for all samples. This threshold is suggested because the square of a decimal fraction begins to depart substantially from the original fraction below a value of 0.95, and the correlation between allele effects between two GWAS is approximately equal to the square of the correlation between trait values. For prospective individuals in a GWAS, only those with accurately measured trait values should be genotyped, such as those that have highly correlated trait measurements.

## Methods

### Samples

The samples on which these analyses were based have been reported previously [20-23]. Briefly, in the Genetic Correlations Experiment of the Cooperative Research Centre for the Cattle and Beef Industry (Beef CRC) there were 9,150 animals with DNA samples and phenotypic measurements from 7 pure breeds (Angus, Hereford, Murray Grey, Shorthorn, Brahman, Belmont Red and Santa Gertrudis) and animals obtained by crossing these breeds to Brahman dams. These were bred from 428 sires with a range of sibships from 1-95. DNA of 940 of these Beef CRC cattle had been used for the GWAS and the selection of these animals was described in detail [10]. These animals form the bulk of the sample reported previously in studies of residual feed intake (RFI) [20,23]. The breed composition of the sample consisted of 220 Angus, 146 Hereford, 55 Murray Grey, 81 Shorthorn, 78 Brahman, 165 Belmont Red, 126 Santa Gertrudis, 25 Taurine-Brahman crossbred and 44 Tropical Composite-Brahman crossbred animals.

In cattle in Australia, subcutaneous fat thickness measurements are taken at the P8 position, which is aligned with the crest of the 3^rd ^sacral vertebra, using a cut and measure procedure with a specially designed ruler with 1 mm gradations (http://www.ausmeat.com.au/industry-standards/meat/beef.aspx, Beef & Veal Language). In this study we distinguish between the trait and the measurement of the trait. Here, P8 subcutaneous fat thickness is the trait and it was measured twice. The usual measurement is the warm dressed carcass measurement and is taken by an accredited AUSMEAT inspector and was called P8FAT in this study. There were 8,653 animals with P8FAT measurements. In addition to this measurement, the animals were also measured at the same location using the same instrument within 24 hours by a team of trained meat scientists in the chiller [7] and is called CHILLP8 in this study. Of the animals with P8FAT measurements, there were 8,139 animals with CHILLP8 measurements from undamaged carcasses after the hide was removed. Fat thickness may change to some extent on cooling so the regression of one measurement on the other may not be 1. Although it might be tempting to view CHILLP8 and P8FAT as different traits, i.e., that there is a biological difference between them, the effects of chilling appear more to be environmental than biological. For example, several random factors affect the speed of chilling, such as location of the carcass in the chiller, length of time in the chiller, the differences between chillers, and a small contribution from differences in fatty acid composition of the fat between individuals, affected by diet and the genetics of the animal [24-26]. Furthermore, an effect of chilling is that fat would be more easily seen on chilled carcasses but warm carcasses are not affected by differences in degree and speed of chilling. Of course, irrespective of the temperature at which the sample is measured, fat layers are not uniformly thick and may also be slightly crenulated, so measurements may differ because of the exact location of the ruler on the tissue. These are not genetic but environmental aspects of the measurement of the trait, so they should not affect the apparent location of the genes affecting the trait. Nevertheless, differences in the measurement of fat thickness itself should merely reflect the differences obtained when two groups measure the same trait in the same way on the same animals at different times and under different conditions, and these should not affect the genetic propensity of the animals to develop a layer of subcutaneous fat.

### Analysis

In total, 940 animals with P8FAT thickness measurements, of which 888 had CHILLP8 measurements, had been genotyped previously using the Illumina Bovine SNP50 Array [27] consisting of 54,001 SNPs [10]. The DNA samples were genotyped by Illumina Inc (Hayward, California) who performed the initial quality control. Genotypes were analyzed for 10%GC scores, call rates, call frequency, cluster separation, and deviations from Hardy-Weinberg Equilibrium using the Genome Studio Software version 1.0. In addition, tests of repeated genotyping of the same animal were included but the identity of the repeat was unknown to the genotyper, and included individuals of known pedigree.

In this analysis the same analytical model and software was used to analyse the associations. In brief, analyses were performed using a mixed model implemented through the software ASReml [28] where the trait ~ mean + fixed effects + SNP genotype + animal + error. Animal and error were treated as random effects. The fixed effects were herd of origin nested in breed, and sex (s) and slaughter group (sg) concatenated to form ssg [10,29]. Five generations of pedigree information was used to construct a numerator matrix defining the relationships between animals. The SNP genotype was coded as number of copies of a reference allele consisting of 0, 1 and 2, so that the association was a regression of the phenotype on number of copies of an allele, and the t-test of the regression coefficient over its standard error was evaluated for significance. Each SNP was analysed one at a time using this model. A SNP array perforce tests a large number of SNP, not all of which are independent. The significance threshold of α = 0.001 was used in the GWAS to limit the number of SNP to be considered. In GWAS, due to the number of tests, the more relaxed the threshold in the discovery sample the more false positives will be discovered. The more stringent the significance threshold the more likely it is that real genetic effects of small size will be overlooked due to sampling effects. The false positive rate FPR = E_p_/O_p _where E_p _is the expected number of SNP with *P *values below a particular significance threshold, given the number of SNP in the panel and assuming that all tests are independent, and O_p _is the observed number of SNP with *P *values below that same threshold. The observed distribution of t-tests was compared to the theoretical distribution in a quantile-quantile (Q-Q) plot using the R program software [30] downloaded from http://www.r-project.org/. The raw phenotypes, adjusted phenotypes and allele regression coefficients for different models were compared by calculating Pearson correlation coefficients.

### Univariate and bivariate data trimming

Two forms of trimming of the data were performed. Univariate trimming was used to remove values from the top and bottom 5% of the distribution. Univariate trimming is a typical method to remove outliers or obviously incorrectly measured individuals [31] and mild 'winsoring' has been used in GWAS analyses [17]. As there were two independent measures of the phenotype in this study, bivariate trimming was used to remove widely divergent estimates of the phenotype. The difference between measurements was diff1 = (y_chillp8 _- y_p8fat_)^2^. A second method, diff2 = (y_chillp8 _- y_p8fat_)^2^/0.5(y_chillp8 _+ y_p8fat_), in which the difference between measurements is scaled by the size of the measurement, yielded essentially the same list of samples, so only diff1 was used in this study. Animals that had extreme trait values either due to univariate or bivariate trimming were excluded from data sets and the GWAS were rerun.

### Trait transformation from quantitative to discrete

To determine how important the misclassification would be if discrete phenotypes were analysed, the fat thickness measurements were treated as if they were a threshold trait. It has been theorised that many discrete traits represent an underlying quantitative trait [32] and when the quantitative underpinning reaches a particular value the individual is classified as affected. In practical terms this might apply to the classification of traits such as obesity in humans, based on height and weight, or schizophrenia based on a cumulative score for a set questionnaire, where a classification threshold needs to be crossed for an individual to be classified as affected. To generate discrete values, adjusted trait values > 1 s.d. above the mean were classified as affected. The adjusted trait values were obtained by analysing the raw data using the model specified above without including SNP information. This resulted in 120 out of 888 animals for CHILLP8 and 128 for P8FAT. The 8 extra P8FAT animals closest to the threshold were classified as unaffected to ensure that the number of 'affected' and 'unaffected' samples were the same, although the identities of the affected samples may be different for each measurement. The sample animals excluded by bivariate trimming at diff1 < 4 and diff1 < 36 respectively were removed from the data set and GWAS for all sets of discrete phenotypes were performed. Tests for trends of affected status on number of copies of alleles were performed [33].

## Abbreviations

BTA14: Bos taurus chromosome 14; CHILLP8: Rump fat thickness measured in millimetres on the chilled carcass at the P8 position by trained meat scientists; diff1 the squared difference between CHILLP8 and P8FAT; FPR: false positive rate; GWAS: Genome wide association study; P8: position aligned with the crest of the 3^rd ^sacral vertebra; P8FAT: Rump fat thickness measured in millimetres on the warm carcass at the P8 position by AUSMEAT inspectors; Q-Q plot: quantile quantile plot of the observed distribution of a statistic against the theoretical distribution of the same statistic; QTL: quantitative trait locus; SNP: single nucleotide polymorphism

## Gene abbreviations

*CHCHD7*: coiled-coil-helix-coiled-coil-helix domain containing 7; *LOC787404*: similar to ribosomal protein S20; *LYN*: v-yes-1 Yamaguchi sarcoma viral related oncogene homolog; *MOS*: v-mos Moloney murine sarcoma viral oncogene homolog; *NSMAF*: neutral sphingomyelinase (N-SMase) activation associated factor; *PENK*: proenkephalin; *PLAG1*: pleiomorphic adenoma gene 1; *RPS20*: ribosomal protein S20; *SDR16C5*: short chain dehydrogenase/reductase family 16C, member 5; *TGS1*: trimethylguanosine synthase 1; *TMEM68*: transmembrane protein 68; *TOX*: thymocyte selection-associated high mobility group box; *XKR4*: XK, Kell blood group complex subunit-related family, member 4

## Authors' contributions

WB planned and performed the analyses, and drafted the manuscript.
